# Therapeutic Potential of Luteolin on Cancer

**DOI:** 10.3390/vaccines11030554

**Published:** 2023-02-27

**Authors:** Melisa Çetinkaya, Yusuf Baran

**Affiliations:** Department of Molecular Biology and Genetics, Faculty of Science, İzmir Institute of Technology, İzmir 35430, Turkey

**Keywords:** flavonoids, Luteolin, anticancer, apoptosis, cell cycle regulation, angiogenesis, metastasis, combination therapy, nanodelivery systems, miRNAs

## Abstract

Cancer is a global concern, as the rate of incidence is increasing each year. The challenges related to the current chemotherapy drugs, such as the concerns related to toxicity, turn to cancer therapeutic research to discover alternative therapy strategies that are less toxic to normal cells. Among those studies, the use of flavonoids—natural compounds produced by plants as secondary metabolites for cancer therapy—has been a hot topic in cancer treatment. Luteolin, a flavonoid that has been present in many fruits, vegetables, and herbs, has been identified to exhibit numerous biological activities, including anti-inflammatory, antidiabetic, and anticancer properties. The anticancer property of Luteolin has been extensively researched in many cancer types and has been related to its ability to inhibit tumor growth by targeting cellular processes such as apoptosis, angiogenesis, migration, and cell cycle progression. It achieves this by interacting with various signaling pathways and proteins. In the current review, the molecular targets of Luteolin as it exerts its anticancer properties, the combination therapy that includes Luteolin with other flavonoids or chemotherapeutic drugs, and the nanodelivery strategies for Luteolin are described for several cancer types.

## 1. Introduction

Cancer, a disease characterized by the abnormal growth and proliferation of cells that may also obtain invasive characteristics, is one of the leading causes of death worldwide, and the burden of the disease on the healthcare system is increasing each year. The International Agency for Research on Cancer predicts that the number of new cancer cases globally, which was around 19.3 million in 2020, is estimated to increase to approximately 30.2 million by 2040 [1]. Despite the extensive research and knowledge on this disease, the currently available treatment options still possess safety and effectiveness problems that affect therapy outcomes and patient compliance. Considering the high predicted incidence rate and the hitherto debilitating loss of human lives, the need to identify and develop novel, efficient, and non-toxic therapeutic drugs for cancer treatment is undeniable [2].

For centuries, the plant kingdom has been an excellent source of natural therapies in the form of herbal extracts used to treat benign and malignant neoplasms. In the past, plant-derived bioactive compounds’ structural and mechanistic characteristics became subjects of intensive research to prevent or mitigate diseases such as inflammation, cardiovascular diseases, neurodegenerative diseases, and in particular, cancer, and extensive research in preclinical settings was initiated [3]. Among these studies, the screening program for anticancer properties of plant-derived compounds commenced by the United States National Cancer Institute (NCI) in 1960 holds an important place. This study identified several novel natural chemicals, including paclitaxel, vincristine, and vinblastine, that have essential roles in clinical settings as chemotherapy drugs, and research on natural anticancer medicines has opened up the way for using structures that nature provides as plants synthesize critical agents used to develop efficient anticancer therapeutics [4].

Among plant-derived bioactive compounds, more than 10,000 flavonoids have been identified and found to be distributed in various ranges of plants, including dietary plants and herbs such as green tea, eggplants, citrus fruits, cacao, and many others. Flavonoids are the polyphenolic secondary metabolites of plants, and they are categorized into six main subcategories: isoflavones, flavanols, flavanones, flavonols, flavones, and anthocyanins, which are commonly included in the diet of humans. Intense research revealed that they play several beneficial roles in human health, including antihypertensive, anti-inflammatory, antiviral, antioxidant, neuroprotective, and anticancer properties [5,6]. Remarkably, the anticancer properties of flavonoids have been researched extensively in both in vitro and in vivo studies and have been found to be primarily related to flavonoids’ ability to regulate oxidative stress inside cells [7]. 

Luteolin (3′,4′,5,7-tetrahydroxyflavone), one of the most extensively researched flavonoids, is a flavone that is widely present in many plant species, particularly in vegetables and fruits such as carrots, celery, onion leaves, broccoli, parsley, sweet bell peppers, and chrysanthemum flowers [8,9]. It is a prevalent natural compound in Chinese traditional medicine, in which plants rich in Luteolin have been widely used to treat diseases such as inflammatory disorders, hypertension, and cancer [10]. Many studies highlighted the multiple biological effects of Luteolin, such as its antiallergy, anti-inflammatory, antidiabetic, neuroprotective, and anticancer properties, and biochemically, based on its chemical structure, it can function as an antioxidant [11]. As an antioxidant, Luteolin and its glycosides can scavenge free radicals caused by oxidative damage and chelate metal ions [12,13]. Additionally, they can inhibit the activity of pro-oxidant enzymes that can cause the production of free radicals. Luteolin and its glycosides can cause the induction of antioxidant enzymes [14]. The biological effects of Luteolin were found to be possibly attributed to each other functionally; for example, the anti-inflammatory function of Luteolin might have been related to its anticancer properties [15]. Luteolin has been developed as a health food for commercial use and has been included in cosmetic products, considering its safety profile and various biological properties. The safety profile of Luteolin has been proven by its non-toxic side effects, as the oral median lethal dose (LD50) was found to be higher than 2500 and 5000 mg/kg in mice and rats, respectively, which was shown to be equal to approximately 219.8−793.7 mg/kg in humans [16].

For years, plants have been used as complementary therapies or dietary agents to manipulate cellular signaling. In light of this, many recent studies showed that among the plant-based compounds, Luteolin could suppress the process of carcinogenesis by altering different cellular events in cancer cells, promoting apoptosis; inducing cell cycle arrest; thus causing perturbation in cell cycle progression; and inhibiting proliferation, migration, and invasion of cancer cells [17]. The anticancer properties of Luteolin have been demonstrated in various cancer types in preclinical settings, and these anticancer effects were found to be regulated through its interaction with different molecular target sites and regulation of several signaling pathways in cancer cells [8,18]; however, no clinical studies of Luteolin have been conducted for cancer treatment yet [18].

This review article describes Luteolin’s structure, natural sources, physiochemical properties, and pharmacokinetics profile, and the central focus of this review is the anticancer activity of Luteolin, which is described for different cancers, including breast, lung, colon, liver, gastric and prostate cancers, and glioblastoma. In particular, this review focuses on the current knowledge of the therapeutic ability of Luteolin against these cancer types, as well as detailed mechanisms of action. Additionally, the use of nanodelivery systems for effective delivery and improvement of the pharmacokinetic properties of Luteolin that might encourage its use in clinical settings is discussed. Overall, this review article aims to address the therapeutic potential of a natural compound, Luteolin, for cancer treatment by highlighting the recent findings in various cancer types. 

## 2. Structure, Physiochemical Properties, and the Natural Sources of Luteolin

**Figure 1:**
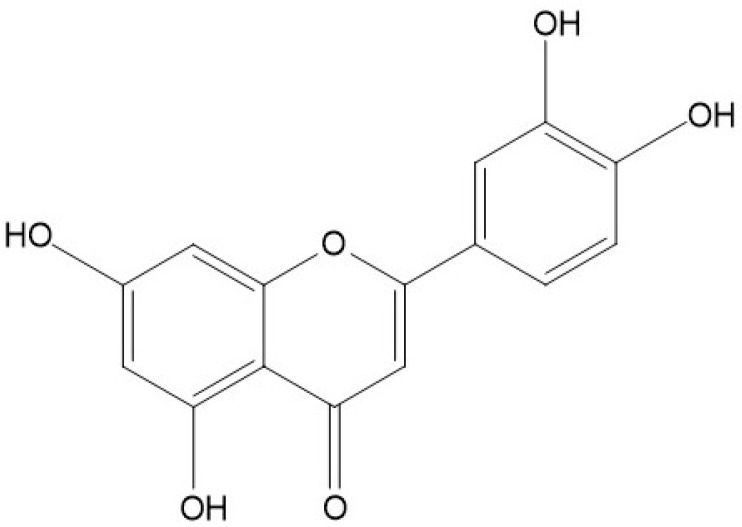
The chemical structure of Luteolin.

Among plant-derived chemical compounds, Luteolin (3′,4′,5,7-tetrahydroxyflavone) is one of the most extensively searched, naturally occurring flavones, a subgroup of flavonoids consisting of a C6-C3-C6 carbon skeleton with two benzene rings linked by a heterocyclic ring (Figure 1). As a chemical, it appears as a yellow crystalline substance that has a molecular formula of C15H10O6 and a molecular weight of 286.24 g/mol, with low water solubility [19,20]. Furthermore, the Luteolin compound is seen as heat stable in nature; thus, interestingly, the molecule cannot be lost during cooking processes [21]. Structure–activity relationship studies revealed that the strong antioxidant activity of Luteolin is due to the hydroxyl groups present at the locations of C5, C7, C3′, and C4′, and the carbonyl oxygen at the C4 site is attributed to its effectiveness against microorganisms. Moreover, the double bond between C2 and C3 has been identified to provide the biocidal activity of Luteolin [15].

Luteolin molecule is widely distributed in plants, mainly as an aglycone molecule that does not have a sugar moiety and as a glycoside molecule (the glycoside form of Luteolin is called LUT-7-O-glucoside or LUT-7G) with a sugar moiety, glucose being the major one that is bound to it [14]. The difference between the aglycone and the glycoside forms of Luteolin mainly lies in its chemical structure; in glycoside form, sugar moieties are attached via one or more hydroxyl groups. The LUT-7-O-glucoside is the most common Luteolin compound introduced in diets comprising foods based on plants and beverages, such as green tea, coffee, nuts, apples, oranges, pomegranates, lemons, grapes, oranges, lettuce, spinach, seaweed, oregano, parsley, thyme, and dark chocolate [9,22]. Additionally, when the activity of the Luteolin (the aglycone form) and the LUT-7-O-glucoside were compared, the aglycone form demonstrated more potent anti-inflammatory, antioxidant, and antidiabetic activities compared to the LUT-7-O-glucoside form [23,24].

## 3. Pharmacokinetics of Luteolin

The knowledge of the pharmacokinetics of Luteolin holds an important place in the understanding and clarification of the relationship between the in vitro activities and in vivo actions of Luteolin. In general, flavonoids, either in free form or glycosylated form, are absorbed from the intestinal tract and metabolized to glucuronide or sulfate conjugates [25]. Chen et al., 2007 found that after oral administration of Luteolin from the Chrysanthemum morifolium extract, it was absorbed rapidly in rats, with the level of Luteolin reaching the highest peak in plasma at 1.1 h after the dosing [26]. Supporting the previous studies’ findings, Yasuda et al., 2015 used a high-performance liquid chromatography (HPLC)−electrochemical detection (ECD) system that showed the free Luteolin in the plasma rapidly increased after 0.5 h post-administration in rats and reached the peak level at 1 h, demonstrating that the Luteolin is absorbed efficiently after oral ingestion [27]. Furthermore, regarding the absorption of the glycosylated form of Luteolin, Shimoi et al., 1998 found that Luteolin in glucoside form was primarily absorbed after its hydrolysis to Luteolin in the free form [28]. Later, Yin et al., 2013 demonstrated that rather than the hydrolysis reaction by lactase phlorizin hydrolase (LPH) and enterobacteria, the Luteolin glucoside could also be absorbed by the sodium–glucose co-transporter 1 (SGLT1) located on the surface of intestinal cells [29]. 

After intestinal absorption, the more significant part of Luteolin is conjugated—in other words, metabolized to other compounds—and a small amount of Luteolin was identified in the urinary and fecal excretion, as Chen et al. 2007 demonstrated in their studies [26]. The bioactivities of Luteolin and its glycosylated form have been related mainly to its metabolite, as Kure et al. 2016 showed in their studies that the Luteolin glucuronides, particularly luteolin-3′-O-glucuronide, exhibited the active Luteolin compound that demonstrated its anti-inflammatory effect in rat studies [30]. Moreover, the pharmacokinetics study conducted in rats by Wang et al. 2017 reported that Luteolin’s metabolites were mainly catalyzed by the UDP-glucuronosyltransferases (UGTs) and catechol-O-methyltransferases (COMTs), as glucuronidation and methylation are considered two critical pathways in the Phase II metabolism and the metabolic disposition of Luteolin and the Luteolin-7-O-glucoside [31]. In the study of Shimoi et al. 1998, in rats and humans, the group identified free Luteolin, its conjugates, and methylated conjugates in the plasma of rats after the dosing. The presence of free Luteolin in the plasma demonstrated that a portion of Luteolin might be able to escape from the intestinal conjugation or the hepatic sulfation/methylation, which was also supported by the presence of free Luteolin and its monoglucuronide in the human serum after Luteolin’s ingestion [28].

One major problem related to the use of flavonoids for therapeutic purposes is their low bioavailability. As flavonoids are polyphenolic compounds, they have a bulky structure that restricts their permeability through the lipoidal cell membrane. Additionally, their low water solubility is a barrier that limits their absorption into the systemic circulation for reaching the wanted plasma levels for the therapeutic action; thus, the bioavailability problem should be taken care of to increase the therapeutic efficiency and potency of these plant-derived chemicals [32,33]. As described by previous pharmacokinetics studies, Luteolin is rapidly absorbed after oral administration; within 30 min, the concentrations of the drug can be detected in the plasma, but its proportions are meager and are rapidly excreted from the kidneys [34]. The extent of the systemic absorption of Luteolin is poor due to its low solubility in water. Furthermore, Luteolin has been identified to undergo extensive pre-systemic metabolism, as the study of Chen et al., 2007 demonstrated the absorption of Luteolin in the small intestine, where they measured the amount of Luteolin in the form of aglycone. This was determined to be very small compared to the amount of total Luteolin glucuronides. This limits the use of Luteolin as a potent and efficient therapeutic molecule [26].

To date, different pharmaceutical studies have been conducted to enhance the solubility and bioavailability of Luteolin by focusing on the main idea of retarding the degradation in the blood to extend the circulation time of Luteolin [35,36,37]. For instance, Khan et al., 2016 used the phospholipid complex to enhance Luteolin’s bioavailability and efficacy in treating inflammatory liver damage [37]. They found that the phospholipid complex of Luteolin demonstrated an increment in relative in vivo bioavailability to 535.31% of Luteolin without phospholipid complex [37]. On the other hand, in their studies, Qing et al. 2017 used different copolymer micelles to enhance the solubility and in vitro release of Luteolin and found that the Luteolin loaded to mPEG5K-PCL10K copolymer micelle system demonstrated higher stability and encapsulation efficiency [36]. In another study, Dang et al., 2014 used a nanoparticle drug delivery system to enhance Luteolin’s bioavailability and pharmacokinetics profile for in vitro and in vivo studies [35]. They found that the Luteolin-loaded nanoparticles demonstrated approximately five times higher bioavailability than the free Luteolin. Additionally, their investigation revealed that using the particular nanoparticle system caused an increase in the plasma Luteolin concentration [35]. In addition, as described previously, another factor that results in the poor bioavailability of Luteolin is the extensive glucuronidation of Luteolin by enzymes such as uridine diphosphate glucuronosyltransferases 1As (UGT1As), which limits its clinical applications [38]. Recently, in their study, Wu et al. 2022 demonstrated that Resveratrol, which functions as the inhibitor of UGT1A1 and UGT1A9, significantly improved the bioavailability of Luteolin by decreasing the major glucuronidation metabolite in rats. Their findings also referenced combining Luteolin and Resveratrol to treat liver diseases [38].

## 4. Anticancer Properties of Luteolin on Different Cancers

**Figure 2:**
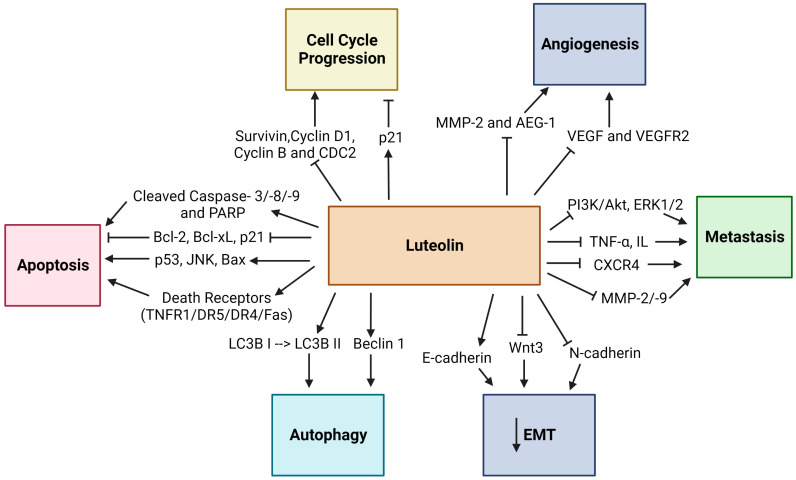
Summary of Luteolin’s regulation of apoptosis, metastasis, angiogenesis, autophagy, cell cycle progression, and EMT in cancer cells. Luteolin was discovered to promote apoptosis of different cancer cells by increasing Death receptors, p53, JNK, Bax, Cleaved Caspase-3/-8-/-9, and PARP expressions and downregulating antiapoptotic proteins such as Bcl-2 and Bcl-xL. It can also inhibit the progression of the cell cycle by downregulating proteins involved in cell cycle progression, including Survivin, Cyclin D1, Cyclin B, and CDC2, and upregulating p21 expression. Luteolin was found to suppress angiogenesis in cancer cells by inhibiting the expression of some angiogenic factors, such as MMP-2, AEG-1, VEGF, and VEGFR2, and inhibit metastasis by inhibiting several proteins that function in metastasis, such as MMP-2/-9, CXCR4, PI3K/Akt, ERK1/2. It can promote the conversion of LC3B I to LC3B II and upregulate Beclin1 expression, thereby causing autophagy induction in cancer cells. Additionally, Luteolin was identified to suppress the epithelial to mesenchymal transition by upregulating E-cadherin and downregulating N-cadherin and Wnt3 expressions.

**Figure 3:**
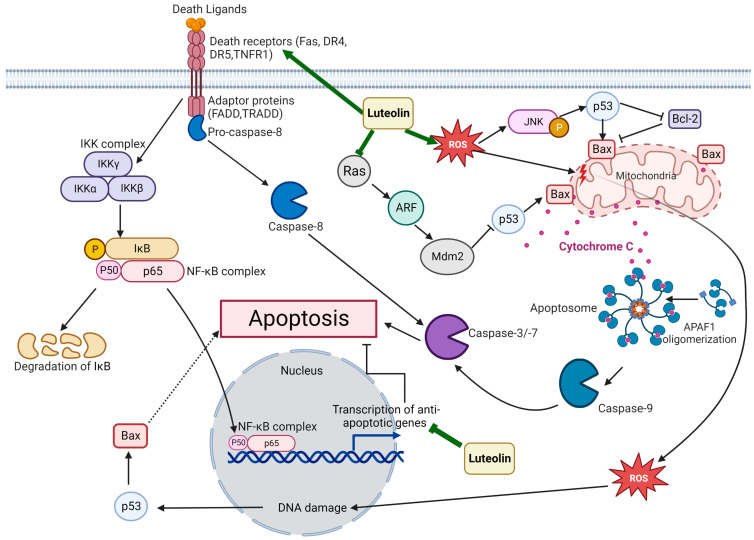
Summary of the possible mechanisms of apoptosis induction by Luteolin in cancer cells. Luteolin has been identified to regulate both extrinsic and intrinsic apoptotic pathways. Luteolin can trigger the extrinsic apoptosis pathway by promoting the expression of death receptors and their downstream factors, and it has been identified to suppress other death receptor signaling pathways contributing to cell survival. It can regulate the intrinsic apoptosis pathway by regulating mitochondrial membrane potential, cytochrome c release, and inhibiting the expression of antiapoptotic proteins such as Bcl-2. Additionally, Luteolin can inhibit Mdm2, which is activated by Ras, and Mdm2 expression is found to trigger the degradation of the tumor suppressor p53. p53 regulates apoptosis by promoting pro-apoptotic protein Bax expression and decreasing Bcl-2 levels. The direct regulation of apoptosis by Luteolin has been identified by modulating DNA damage that is induced by reactive oxygen species (ROS), where DNA damage signaling has been determined to promote p53 production and activity. Arrows designate activation (↑) and inhibition (⊥) of the molecules, and green arrows represent activation (↑) and inhibition (⊥) of the molecules by Luteolin.

**Figure 4:**
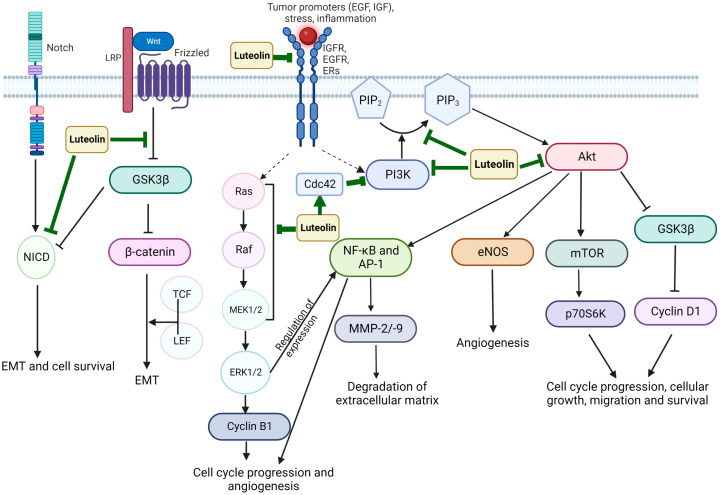
Summary of Luteolin’s regulation of several cancer signaling pathways in different cancer types. Luteolin can block the Notch intracellular domain (NICD) that is created by the activation of the Notch, resulting in the removal of the intracellular domain of the membrane-bound Notch via an unknown mechanism. Wnt/β-catenin can result in inhibition of the GSK3β activity after activation, and Luteolin can inhibit the phosphorylation of the GSK3β induced by Wnt, resulting in the prevention of GSK3β inhibition. Additionally, Luteolin can inhibit the activities of some receptor tyrosine kinases (RTKs) such as IGFR, EGFR, and ERs and their downstream effector molecules that result in the inhibition of several cellular processes contributing to cancer cell progression and development. Arrows designate activation (↑) and inhibition (⊥) of the molecules, and green arrows represent activation (↑) and inhibition (⊥) of the molecules by Luteolin.

The anticancer properties of Luteolin have been related to its ability to inhibit proliferation, metastasis, and invasion of tumor cells and angiogenesis by various mechanisms, including the suppression of kinases, promotion of apoptosis, regulation of the tumor cell cycle, and reduction in transcription factors (Figure 2) [17]. The induction of apoptosis has been linked with Luteolin’s anticancer properties, which involve DNA damage, regulation of redox, and protein kinases in inhibiting cancer cell proliferation (Figure 3) [39]. Moreover, Luteolin has been demonstrated to have an inhibitory effect on the proliferation of tumor cells with IC50 values in the range between 3–50 µM in vitro [40], and a significant decrease in the tumor volume was observed in mice that were fed with a diet containing Luteolin at 50–200 ppm for six weeks [41]. In this section, the anticancer activity of Luteolin in different cancer types will be described, along with the various signaling pathways affected by Luteolin, as well as its targets (Figure 4). Table 1 summarizes the critical research described throughout this section on Luteolin’s anticancer properties, its molecular targets/or signaling pathways, and its effects on different model systems.

### 4.1. Luteolin and Colon Cancer

Thus far, extensive research has been conducted to demonstrate the anticancer properties of Luteolin on colon cancer and the molecular pathways that Luteolin targets to exhibit its anticancer activities. In earlier studies, the anti-inflammatory and antioxidant abilities of Luteolin have been identified as responsible for the effectiveness of Luteolin in colon cancer and the complications associated with it, particularly the decreasing effect on the expressions of inducible nitric oxide synthase (iNOS) and cyclooxygenase-2 (COX-2) [42]. Additionally, the suppression of the matrix metalloproteinase-2 (MMP-2) and MMP-9 expression by Luteolin is another mechanism that has been shown to be related to the anticancer effects of Luteolin on colon cancer by inhibiting angiogenesis, a crucial factor for tumor progression [43]. Moreover, in the study conducted by Pandurangan et al., 2014, Luteolin has been identified to increase the expression of nuclear factor erythroid 2-related factor 2 (Nrf2), which is a crucial transcription factor with anticarcinogenic properties related to the Nrf2/antioxidant responsive element (ARE) pathway. This is an essential pathway in the regulation of intracellular redox status in mice bearing colorectal cancer (CRC) induced by Azoxymethane (AOM) [44].

The effect of Luteolin on apoptosis and growth arrest was studied in different colon cancer cell lines and mouse models. As shown by Pandurangan et al. 2013 Luteolin was identified to cause the inducement of growth arrest via inhibition of Wnt/β-catenin/glycogen synthase kinase-3 beta (GSK-3β) signaling pathway and caused promotion of apoptosis in a Caspase-3 mediated manner in HCT-15 colon adenocarcinoma cell line [45]. In another study by Pandurangan and Ganapsam.,2013, Luteolin was shown to decrease the lysosomal enzymes’ activities and induce apoptosis by reducing the expression of antiapoptotic protein Bcl-2 while driving an increment in the pro-apoptotic protein Bax and Caspase-3 levels in AOM-induced colon cancer in aBalb/C mouse model [46]. Moreover, Luteolin has been shown to induce apoptosis through activation of the mitochondria-mediated caspase pathway in HT-29 colon cancer cell line and caused loss of the mitochondrial membrane action potential, enhanced levels of mitochondrial calcium (Ca2+), increased Bcl-2-associated X protein (Bax) and decreased B-cell lymphoma 2 (Bcl-2) expressions, and caused an increment in the active Caspase-9 and Caspase-3 levels [47]. Additionally, the same study identified that the apoptotic effect of Luteolin was intervened by the activation of the Mitogen-Activated Protein Kinase (MAPK) signaling in human colon cancer cells [47]. A recent study by Kang et al. 2018 examined the underlying molecular mechanisms of the apoptotic effect of Luteolin mediated by the DNA methylation of the Nrf2 promoter and the interaction of Nrf2 and a well-known tumor suppressor, p53, in human colon cancer cells [48]. The study revealed that Luteolin demonstrated its anticancer effects on HT-29 and SNU-407 colon cancer cell lines by promoting apoptosis, increasing the Nrf2 transcription that is influenced by the enhancement in the DNA methylation of Nrf2 promoter, and by causing an increment in the interaction between Nrf2 and p53. This resulted in increased expression of antioxidant enzymes and proteins related to apoptosis [48].

Furthermore, in a study by Yoo et al., 2022, Luteolin has been shown to inhibit the HCT116 colon cancer cells’ growth through p53-dependent regulation of apoptosis and cell cycle arrest, regardless of the autophagy induction [49]. When the effect of Luteolin on cell cycle arrest was investigated in human colon cancer cells, Chen et al. 2018 found that Luteolin caused cell cycle arrest at the G2/M phase, followed by the induction of apoptosis in LoVo human colon cancer cells [50]. Further molecular mechanism studies showed that the Luteolin demonstrated an inhibitory effect on LoVo cells’ proliferation through inhibition of the cell cycle arrest at the G2/M phase transition, inactivating cyclin B1/cell division cycle 2 (CDC2), followed by the induction of apoptosis by cytochrome c- and deoxyadenosine triphosphate-mediated activation of apoptotic protease activating factor 1 [50]. In a study by Song et al., 2022, Luteolin was found to suppress cell proliferation, cause cell cycle arrest, and induce DNA damage and apoptosis in CRC cells through modulation of the MAPK pathway, while Luteolin inhibited the tumor growth in the CRC xenograft model in vivo [51]. Additionally, in the same study, Song et al. 2022 demonstrated that Luteolin enhanced the effect of one of the most effective chemotherapy drugs, cisplatin, on CRC cells, as the combination of Luteolin and cisplatin caused a significant decrease in cell survival and increased the rate of apoptosis of HCT-116 and HT-29 cells, compared with the only cisplatin treatment [51].

Interestingly, Jang et al., 2019 demonstrated that a high dose of Luteolin application negatively affected the oxaliplatin-based chemotherapy in a p53-dependent manner [52]. They suggested that the flavonoids with Nrf2-activating ability might interfere with the chemotherapeutic efficacy of anticancer agents in CRC cells with functional p53 protein [52]. In a recent study, on the other hand, Aromokeye and Si, 2022 examined the synergistic inhibitory effect of the combination of Luteolin with another phytochemical, Curcumin, on colon cancer cells and found that the combination of Luteolin and Curcumin synergistically inhibited the proliferation of CL-188 and DLD-1 cells and the tumor growth in the CL-188 cells-derived xenograft mice [53]. In contrast, the individual Luteolin and Curcumin did not exhibit anticancer effects at the dosages selected in vitro and in vivo. This combination’s synergistic anti-colon cancer effect was found to be related to the regulation of Notch1 and transforming growth factor- beta (TGF-β) pathways and the induction of necrosis in cell lines and tumors [53].

In their study, Jiang et al., 2022, examined the therapeutic potential of Luteolin in colon cancer, with a specific focus on its effect on the tumor microenvironment [54]. They revealed that Luteolin inhibited the growth, migration, and invasion potential of SW620 and SW480 colon cancer cells caused by M1 polarization by acting on the interleukin-6 (IL-6)/signal transducer and activator of transcription 3 (STAT3) pathway [54]. As the IL-6/STAT3 pathway is an important pathway that regulates the progression of colon cancer, inhibition of this pathway by Luteolin provided new insight into the therapeutic potential and mechanisms of Luteolin for colon cancer treatment [54]. Finally, Yao et al., 2019, in their study, aimed to investigate the roles of micro RNAs (miRNAs) in treating CRC cells with Luteolin and found that Luteolin inhibits the migration and invasion of the CRC cells by regulating the miR-384/pleiotrophin axis, suggesting that miR-384 and pleiotrophin can be important targets for treating CRC [55].

### 4.2. Luteolin and Lung Cancer

Luteolin has been identified as a potential therapeutic agent for lung cancer due to its ability to manipulate multiple targets responsible for lung cancer progression and development. In earlier studies, Meng et al. 2016 demonstrated in human non-small cell lung cancer (NSCLC) cell line A549 that Luteolin exhibited a substantial anticancer effect by inducing apoptosis and inhibiting migration of NSCLC cells [56]. The induction of apoptosis by Luteolin was associated with an increment in the activation of Caspase-3 and Caspase-9, decreased Bcl-2 and increased Bax expressions, and the phosphorylation of mitogen-activated protein kinase kinase (MEK) and the downstream kinase of MEK, which is extracellular signal-regulated kinase (ERK), as well as Akt activation. Overall, their study highlighted that the MEK-ERK signaling pathway exhibited a crucial role in intervening in Luteolin’s pro-apoptotic and antimigration effects in NSCLC cells [56]. On the other hand, in an extensive molecular mechanistic study, Luteolin was identified to induce apoptosis by modulating both intrinsic and extrinsic pathways, which were suppressed by z-Val-Ala-Asp-fluoromethylketone (z-VAD-fmk), a pan-caspase inhibitor, showing that Luteolin was able to trigger caspase-dependent apoptosis in NCI-H460 human NSCLC cells [57]. Park et al., 2013 also found that Luteolin induced apoptosis through phosphorylated eukaryotic initiation factor 2 alfa (eIf2α)/C/EBP homologous protein (CHOP), suggesting that apoptosis induced by Luteolin in NCI-H460 cells could be related to the endoplasmic reticulum (ER) stress [57]. Moreover, they showed that Luteolin could also induce autophagy, and when autophagy was inhibited, the apoptotic cell death was reduced so that the Luteolin-induced autophagy was found to function as a cell death mechanism [57]. In another study, Luteolin demonstrated its anticancer properties in NCI-H460 cells by promoting Sirt1-mediated apoptosis [58]. Recently, Luteolin was determined to inhibit the lung cancer cells’ anchorage-independent colony growth and promote apoptosis and cell cycle arrest at the G1 phase [59]. It decreased the expression of cyclin D1 and enhanced cleaved Caspase-3 levels by downregulating LIM domain kinase (LIMK) 1 signaling-related targets, such as phosphorylated LIM domain kinase (p-LIMK) and p-cofilin in vitro. It also inhibited tumor growth in the lung cancer patient-derived xenograft by decreasing Ki-67, p-LIMK, and p-cofilin expressions [59].

Tumor-associated macrophages (TAMs) play essential roles in cancer progression [60], and Choi et al. 2016 aimed to examine the part of Luteolin in the inhibition of tumor-supporting M2-like phenotype of TAMs by using a murine macrophage cell line RAW 264.7 cells treated with IL-4 [61]. They found that Luteolin inhibited the phosphorylation of STAT6, a primary downstream signal of IL-4, and caused a decrement in the expression of M2-associated genes. They also found that Luteolin caused a decrement in the migration of Lewis lung carcinoma cells in a way that was dependent on chemokine (C-C motif) ligand 2 (CCL2) [61]. As the TAM phenotype plays a vital role in the tumor microenvironment, the inhibitory effect of Luteolin on the monocyte recruitment and migration of cancer cells via suppression of the TAM-secreted CCL2 was suggested as a novel therapeutic strategy for cancer [61]. Immunotherapy is an outstanding therapeutic strategy for cancer treatment. Upregulation of the immune checkpoint molecules is associated with the exhausted phenotype and impairment in the function of cytotoxic T-cells to escape host immunity. By disrupting the interaction between programmed death protein 1 (PD-1) and its ligand programmed death-ligand 1 (PD-L1), immune checkpoint inhibitors can restore the immune system’s ability to fight against cancer cells [62,63]. In a study by Jiang et al. 2021, the effect and the underlying mechanism of Luteolin, Apigenin, and the anti-PD-1 antibody combined with either Luteolin or Apigenin on the PD-L1 expression and anticancer properties were investigated in Kirsten rat sarcoma virus (K-Ras) mutant lung cancer [64]. As a result, it was found that Luteolin and Apigenin dramatically inhibited the growth of lung cancer cells, promoted apoptosis, and decreased the expression of interferon-gamma-(IFN-γ)-induced PD-L1 by suppressing the phosphorylation of STAT3. Moreover, Luteolin and Apigenin were found to demonstrate potent anticancer activities in vivo in the xenograft models of H358 and Lewis lung carcinoma, and treatment with the monoclonal PD1 antibody increased the T-cell infiltration to tumor tissues. This suggests that combination therapy of PD-1 blockade and Apigenin or Luteolin could have a synergistic effect for NSCLC with K-Ras-mutant [64].

The combination treatment that includes anticancer drugs and radiotherapy to improve the therapeutic efficacy and survival of cancer patients is a fundamental approach to the treatment of NSCLC, as it is for many other cancer types. It is based on the strategy that anticancer drugs implement a different mechanism than radiotherapy and, most importantly, may increase the sensitivity of cancer to the effects of ionizing radiation. Those anticancer drugs are named radiosensitizers [65]. From this perspective, in their study, Cho et al., 2015, investigated the radiosensitizing activity of Luteolin in NSCLC and revealed that in NCI-H460 and NCI-H1299, NSCLC cells, co-treatment of Luteolin and ionizing radiation promoted apoptosis by downregulating Bcl-2 and activating Caspase-3, Caspase-8, and Caspase-9; it also caused induction of phosphorylation of p38 MAPK and reactive oxygen species (ROS) accumulation [66]. Additionally, the same study showed that in the NCI-H460 cell xenograft mice model, the combination of Luteolin and ionizing radiation caused a delay in tumor growth and increased apoptosis compared to control groups; it also suggested that Luteolin can act as a radiosensitizer, promoting apoptosis by inducing p38/ROS/caspase cascade [66]. Tumor necrosis factor-related apoptosis-inducing ligand (TRAIL) is a protein that belongs to the tumor necrosis factor (TNF) family. It induces apoptosis of tumor cells explicitly but does not harm normal cells [67]. Cancer cells have been identified to show extreme sensitivity to TRAIL compared to normal cells. Thus, TRAIL was found to hold a crucial potential as a novel and effective cancer therapeutic, but its effect in terms of therapy is limited because of drug resistance [68]. Wu et al., 2020 discovered that Luteolin was able to enhance TRAIL sensitivity in NSCLC cells by increasing the expression of death receptor 5 (DR5), the receptor of TRAIL, and increasing the dynamin-related protein 1 (Drp1) mediated mitochondrial fission through c-Jun N-terminal kinase (JNK) signaling, suggesting that combination therapy of Luteolin with TRAIL could be an effective strategy for the treatment of NSCLC [69].

Epithelial–mesenchymal transition (EMT) is critical when transforming a benign into a malignant tumor. It is considered a pathological step that promotes cancer progression, especially invasion and metastasis, and Chen et al. 2013 aimed to investigate the role of Luteolin in the invasion/metastasis of lung cancer cells [70]. They found that Luteolin pretreatment of the A549 human lung adenocarcinoma cells prohibited the morphological change and caused downregulation of E-cadherin activated via TGF-β1. Additionally, the study revealed that the activation of the phosphoinositide 3-kinase (PI3K)-Akt-inhibitory subunit of NF Kappa B Alpha (IκBα)-nuclear factor kappa B(NF-κB)-Snail pathway led to a decrease in the E-cadherin level induced by TGF-β1, which was also diminished with the pretreatment of Luteolin [70]. In a more recent study, Masraksa et al. 2020 demonstrated that Luteolin restricted the migration of A549 cells by inhibiting the development of focal adhesion and diminishing the focal adhesion kinase (FAK)-Src signaling [71]. Additionally, Luteolin was found to decrease the expression levels of Ras-related C3 botulinum toxin substrate 1 (Rac1), cell division control protein 42 (Cdc42), and Ras homolog gene family member A (RhoA), which are responsible for the regulation of actin cytoskeleton and cell migration [71].

As increasing numbers of studies have highlighted the critical roles of miRNAs in the development of progression of NSCLC and the anticancer effects of Luteolin by modulating miRNA in various cancer types [72], the modulation of miRNAs by Luteolin in lung cancer has also been extensively researched. In their study, Jiang et al., 2018 found that Luteolin inhibited growth and promoted apoptosis of A549 and H460 cells. Additionally, in an H460 xenograft model, it significantly inhibited tumor growth and cell proliferation and induced apoptosis [73]. Furthermore, they revealed that miR-34a-5p was significantly upregulated in the tumor tissues upon Luteolin treatment, and mouse double minute 4 (MDM4), a potential oncogene responsible for the repression of p53 transcription and induction of its proteasomal degradation, was found to be a direct target of MDM4, as the Luteolin treatment was associated with increased p53 and p21 and decreased MDM4 expressions both in vitro and in vivo. Additionally, when the miR-34a-5p was inhibited in vitro, Bcl-2 and MDM4 expressions were found to be recovered, whereas p53, p21, and Bax expressions were decreased. Thus, it was demonstrated that Luteolin could inhibit tumorigenesis and promote apoptosis of NSCLC cells, upregulating miR-34a-5p by targeting MDM4 [73]. In another study, Luteolin was reported to inhibit the viability, migration, angiogenesis, and invasion of vascular endothelial cells of NSCLC, whereas it upregulated the miR-133a-3p level. When the inhibitor of miR-133a-3p was used, the inhibitory effect of Luteolin on the viability, migration, angiogenesis, and invasion in vascular endothelial cells of NSCLC was counteracted [74]. Moreover, in the same study, Pan et al. 2022 revealed that Luteolin caused the downregulation of proteins associated with migration and invasion (vascular endothelial growth factor (VEGF), MMP-2 and MMP-9), factors related to PI3K/Akt and MAPK signaling pathways. In contrast, inhibition of miR-133a-3p reversed the inhibitory effects of Luteolin [74]. Additionally, Luteolin was found to decrease the purine-rich element binding protein B (PURB), targeted by miR-133a-3p levels in vascular endothelial cells of NSCLC, and when PURB was silenced, the miR-133a-3p levels increased. Further examinations indicated that Luteolin inhibited the migration and invasion of vascular endothelial cells of NSCLC by miR-133a-3p/PURB- mediated MAPK and PI3K/Akt pathways [74].

Apart from the regulation of miRNAs by Luteolin in lung cancer, Zheng et al. 2022 reported that Luteolin could regulate the circular RNAs (circRNAs), specifically circ_0000190, which was found to be upregulated in lung cancer tissues [75]. In their study, they discovered that Luteolin inhibited the viability, colony formation, migration, invasion, and induced apoptosis of lung cancer cells, and the overexpression of circ_0000190 was able to counteract the role of Luteolin in the suppression of lung cancer development [75]. Moreover, in the same study, circ_0000190 was shown to be directly bound with miR-130a-3p. The study revealed that Luteolin suppressed the growth of lung cancer cells, metastasis, and Notch-1 signaling pathway by regulating the circ_0000190/miR-130a-3p axis in vitro and by regulating circ_0000190, Luteolin suppressed the tumor growth of lung cancer in vivo [75].

### 4.3. Luteolin and Prostate Cancer

Luteolin has been identified to have therapeutic and chemopreventive roles in prostate cancer related to Luteolin’s ability to inhibit growth and invasiveness and promote the apoptosis of prostate cancer cells, but also decrease the contraction of the extracellular matrix through different targets and signaling mechanisms [76,77,78,79,80,81]. In highly invasive Du145-III isolated prostate cancer cells, Luteolin decreased the malignancy, vasculogenic mimicry, and anchorage-independent spheroid formation of Du145-III cells. Additionally, Luteolin caused a decrement in the expression of specific cancer stem cell markers and was suggested to be a potential antiangiogenesis and antimetastasis agent for prostate cancer cells [77]. Moreover, in a study by Zhou et al., 2009, Luteolin was shown to inhibit the invasion of PC3 prostate cancer cells by inducing E-cadherin expression, where the induction of the E-cadherin expression by Luteolin was found to occur through the MDM2 protein, as the invasion of PC3 cells by overexpressing MDM2 or knockdown of E-cadherin could be restored after Luteolin treatment [76]. Additionally, in the same study, Luteolin was discovered to inhibit MDM2 by Akt, and overexpression of active Akt resulted in the decrement of Luteolin-induced expression of E-cadherin. Thus, Luteolin was found to regulate E-cadherin through the Akt/MDM2 pathway in prostate cancer [76]. Furthermore, in a study by Pratheeeshkumar et al., 2012, the antiangiogenic activity of Luteolin was further examined in vitro, ex vivo, and in vivo [78]. Their results demonstrated that Luteolin was able to significantly inhibit VEGF-stimulated endothelial cell proliferation, chemotactic migration, invasion, tube formation, and angiogenesis by targeting the VEGFR-2-regulated Akt/ERK/mammalian target of rapamycin (mTOR)/P70S6K/MMPs pathway that caused the suppression of prostate tumor growth and angiogenesis. Luteolin was found to suppress angiogenesis ex vivo, as measured via a chick embryo chorioallantoic membrane (CAM) assay, and in vitro, as measured by a rat aortic ring assay [78]. Furthermore, Luteolin was revealed to inhibit cancer growth by promoting apoptosis and angiogenesis in the human prostate xenograft mouse model and thus reduce the proinflammatory cytokines, such as IL-1β, IL-6, IL-8, and TNF-α production in PC-3 (prostate cancer cells) [78]. In a recent study by Han et al., 2018, Luteolin was identified to suppress the Wnt signaling by upregulating frizzled class receptor 6 (FZD6), the negative regulator of β-catenin transcriptional activity, and causing the inhibition of the stemness of prostate cancer cells [79].

In their study, Tsui et al. 2012 revealed that Luteolin at the concentration of 30 µM was effective against human prostate carcinoma LNCaP cells via promoting apoptosis, upregulating prostate-derived Ets factor (PDEF), and downregulating the androgen receptor (AR) expression [80]. Additionally, Luteolin was found to increase expressions of B-cell translocation gene 2 (BTG2), N-myc downstream-regulated gene 1 (NDRG1), and Maspin, and the transient gene expression assays demonstrated that co-transfecting the PDEF expression vector caused an increment in the promoter activities of BTG2, NDRG1, and Maspin genes. Overall, the study highlighted the importance of Luteolin in blocking prostate-specific antigen expression by downregulating AR expression [80]. In the study of Markaverich and Vijjeswarapu, 2012, Luteolin was found to reduce the expression of several genes in the cell cycle pathway (CCP) and epidermal growth factor receptor signaling pathway (EGFRSP) in PC-3 cells [81]. It stimulated p21 RNA and c-FOS expressions and irreversibly caused G2/M cell cycle arrest. Moreover, they showed that p21 or c-FOS silencing RNAs (siRNAs) dramatically reduced the RNA expression of their corresponding targets but had minor effects on the propagation of cells, and the inhibition of PC-3 cell proliferation was not blocked by either one siRNA or double siRNA [81].

Studies related to the anticancer effects of Luteolin also focused on the combination of Luteolin with other natural compounds or other chemical drugs that can be used as chemotherapeutic agents for the treatment of prostate cancer [82,83,84]. In one of the studies, Wang et al. 2014 aimed to demonstrate the in vivo antimetastatic effects of the combination of pomegranate juice’s natural components: Luteolin, Ellagic Acid, and Punicic Acid in prostate cancer [82]. Their study revealed that Luteolin, Ellagic Acid, and Punicic Acid inhibited growth and metastasis in the C-X-C motif chemokine ligand 12 (CXCL12)/C-X-C chemokine receptor type 4 (CXCR4) axis in human prostate cancer xenograft tumors in severe combined immunodeficiency mice [82]. The combination of the compounds also inhibited the growth and metastasis of allograft tumors of the highly invasive mouse prostate cancer cells that had a deletion of phosphatase and tensin homolog (PTEN) and K-Ras activation. Moreover, this therapeutic strategy combining three compounds resulted in the inhibition of angiogenesis in vivo; this strategy prevented the formation of human endothelial cell tubes in culture, disrupted the endothelial cell tubes that were formed previously, and inhibited the angiogenic factors IL-8 and VEGF, together with their induced signaling pathways in endothelial cells [82]. In a study with a similar perspective, using two plant-derived chemicals for the treatment of prostate cancer, Gray et al. 2014 demonstrated that micromolar combinations of (−)-Epigallocatechin-3-gallate (EGCG) and Luteolin inhibited the TGF-β-induced myofibroblast phenotypes synergistically in prostate fibroblast cell lines, as evidenced by the potentiation of fibronectin expression [83]. Functional studies revealed that EGCG and Luteolin inhibited the extracellular matrix contraction induced by TGF-β, functioning as the enhancer of the tumor cell invasion. Moreover, EGCG and Luteolin were found to inhibit the downstream of the TGF-β-induced signaling, including ERK and Akt activation, respectively; however, only ERK seemed necessary for the TGF-β-induced fibronectin expression. As Rho signaling was known to be required for the TGF-β-induced fibronectin expression, the inhibition of RhoA by EGCG and Luteolin was further investigated and shown [83]. On the other hand, Sakurai et al., 2014 studied the combinatory effect of Luteolin and Gefinitib, a selective tyrosine kinase inhibitor that inhibits the EGFR, but also the kinase activity of cyclin G-associated kinase (GAK), on PC-3 prostate cancer cells [84]. As a result, they found that combined treatment of Luteolin and Gefitinib dramatically reduced the viability of PC-3 cells compared to the effect of each drug alone. Furthermore, they were shown to significantly reduce the kinase activity of GAK, which was found to be overexpressed in hormone-refractory prostate cancer, and inhibition of its kinase activity was suggested as a novel molecular-target therapy. Additionally, they discovered that Luteolin and gefitinib induced the expression of miR-630, which led to the arrest of PC-3 cells’ and again highlighted the importance of miRNAs in the regulation of the cancer progression and development [84]. Another miRNA Luteolin target was found in a study by Han et al., 2016, in which they described that Luteolin was found to inhibit the proliferation and promote apoptosis of prostate cancer cells by downregulating miR-301 on oncogenic miRNA. It achieved this by inducing expression of the death effector domain containing 2 (DEDD2), which is a pro-apoptotic protein [85].

### 4.4. Luteolin and Gastric Cancer

The effective anticancer properties of Luteolin were demonstrated in different experimental models and were found to be related to the regulation of various proteins and signaling pathways. In general, it was found that Luteolin exerted its biological properties by inhibiting Cyclin D1, Cyclin E, Bcl2, MMP-2, MMP-9, N-cadherin, Vimentin, and inducing p21, Bax, and E-Cadherin expressions in gastric cancer cells. Its anticancer effects were achieved by reducing Notch1, p-PI3K, p-AKT, p-mTOR, p-ERK, and p-STAT3 and increasing p-P38 signaling in gastric cancer cells [86]. In an earlier study, the application of Luteolin at 40 mg/kg caused effective inhibition of tumor growth in BGC-823 gastric carcinoma xenografts in mice, and the mechanism of inhibition of tumor growth was suggested to be related to the stimulation of immune response and suppression of VEGF-A and MMP-9 expressions [87]. Moreover, Lu et al., 2015 aimed to demonstrate the antitumor effect of Luteolin in cMet-overexpressing patient-derived xenograft models of gastric cancer, which was an essential step in gastric cancer treatment, as the c-Met overexpression in gastric cancer has been related to a poor prognosis due to the high tumor metastasis and limited therapeutic strategies [88]. These researchers found that Luteolin caused significant inhibition in tumor growth in cMet-overexpressing patient-derived xenograft models, and immunohistochemistry studies revealed that Luteolin significantly decreased cMet, MMP-9, and Ki-67 expressions in tumor tissues. Furthermore, Luteolin caused inhibition of proliferation and invasiveness and promoted apoptosis of c-Met overexpressing gastric cancer cell lines, MKN45 and SGC7901; further studies indicated that Luteolin enhanced the activation of proteins related to apoptosis, such as Caspase-3 and poly (ADP-ribose) polymerase-1 (PARP-1), and caused downregulation of invasion-related protein MMP-9 [88]. Additionally, Luteolin led to decreased expression and phosphorylation of cMet and downstream phosphorylation of Akt and ERK, and the downregulation of phosphorylated Akt levels was found to be independent of cMet [88].

Zang et al., 2017 demonstrated that Luteolin dramatically inhibited the proliferation, invasion, and migration of gastric cancer cells in a dose- and time-dependent manner, induced apoptosis in vitro, and caused tumor growth suppression in vivo [89]. Luteolin application caused a reversion of EMT via shrinkage in the cytoskeleton and increment in the expression of epithelial biomarker E-cadherin and downregulation of the mesenchymal biomarkers N-cadherin, vimentin, and Snail in gastric cancer cells. Additionally, Notch1 signaling was found to be inhibited by Luteolin [89]. In a further study by the same group, Luteolin was shown to dramatically inhibit tube formation of the human umbilical vein endothelial cells (HUVECs) by causing a decrement in cell migration and proliferation [90]. Moreover, in the examinations of gastric cancer cells, Luteolin resulted in the inhibition of vasculogenic mimicry formed by Hs-746T gastric cancer cells and found that a dramatic decrease in the VEGF secretion of Hs-746T cells resulted from the inhibition of Notch1 expression in gastric cancer [90]. Thus, it was suggested that Luteolin inhibited angiogenesis and vasculogenic mimic formation in gastric cancer cells by suppressing VEGF secretion based on the Notch1 expression [90].

miR-34a is an example of a well-researched miRNA therapeutic that functions as a tumor suppressor, which was discovered to be downregulated in various cancer types, including gastric cancer [91]. Wu et al., 2014 found that Luteolin caused a decrement in the Bcl-2, antiapoptotic protein level by upregulating the tumor-suppressor miR-34a expression in gastric cancer cells; thus, for the first time, they revealed that miR-34a pathway held an important place in the Luteolin-induced apoptosis in gastric cancer cells [92]. Further studies showed that as Luteolin caused an increase in the cell viability of the gastric cancer cells, it also caused upregulation of miR-34a, and the artificial Luteolin-resistant gastric cancer cells showed decreased miR-34a expression [93]. Moreover, upregulation of the miR-34a in cells resistant to Luteolin enhanced the sensibility of the cells to Luteolin, and examinations in the mouse xenograft models of gastric cancer revealed that targeting miR-34a was able to mediate susceptibility to Luteolin [93]. In the same study by Zhou et al., 2018, hexokinase-1 (HK1) was found as a direct target of miR-34a in gastric cancer cells and suggested that miR-34a could modulate the susceptibility of gastric cancer cells to Luteolin by targeting HK1 [93]. Additionally, apart from the miR-34a, Luteolin was found to increase miR-139, miR-422a, and miR-107, and decreased miR-21, miR-155, miR-244 and miR-340 levels in gastric cancer cells, but how targeting of these miRNAs by Luteolin affects gastric cancer cell progression has yet to be fully determined [86].

The radiosensitizer effect of Luteolin was identified on the gastric cancer cells. In a study by Zhang et al., 2009, Luteolin was shown to increase the irradiation-induced colonogenic inhibition and Caspase-3 and Caspase-9 activities [94]. Additionally, a decrease in the levels of Bcl-2, VEGF, and hypoxia-inducible factor 1-alpha (HIF-1α), as observed in the Luteolin and radiation treatment in gastric cancer SGC-7901 cells, as well as Luteolin was found to dramatically enhance the radioresponse of human gastric cells transplanted into mice [94]. Furthermore, in a recent study, Luteolin was suggested as a chemosensitizer in gastric cancer as Ren et al. 2020 demonstrated the synergistic effect of Oxaliplatin and Luteolin on the inhibition of the proliferation of gastric cancer cells and induced apoptosis in vitro and showed that Luteolin was able to potentiate the sensitivity of SGC-7901 cells to Oxaliplatin via the cytochrome C/caspase pathway [95]. The effects of the Luteolin on the uptake of Oxaliplatin might be related to increased cleaved Caspase-3 and Bax levels but were also associated with the release of cytochrome C from the mitochondria [95].

### 4.5. Luteolin and Glioblastoma

As Luteolin was discovered to pass the blood–brain barrier due to its lipophilicity, its biological functions in the central nervous system and neurological disorders have been investigated extensively [96]. Additionally, the anticancer properties of Luteolin in brain cancer types, specifically in glioblastoma, were among the hot topics in cancer therapeutics, and the anticancer activity of Luteolin against glioblastoma cells was found to be related to its ability to inhibit cell growth, promote apoptosis, and decrease invasion and migration of glioblastoma cells. Luteolin was found to effectively reduce the viability of A172 and U-373MG glioblastoma cells in a dose- and time-dependent manner. In their study, Lee et al., 2021 demonstrated that treatment of A172 and U-373MG cells with Luteolin at concentrations higher than 100 µM caused nuclear fragmentation, morphological change related to apoptosis, and fragmentation of Caspase-3 and PARP, which were apoptosis-related factors [97]. Additionally, treatment of cells with Luteolin induced autophagy, and importantly, when Luteolin-induced autophagy was inhibited, apoptosis was found to be promoted in A172 and U-373MG cells. Thus, it was suggested that Luteolin-induced autophagy was a survival signal and was considered to interfere rather than promote a call that can lead to apoptosis in glioblastoma cells [97]. In another study, the antiproliferative effects of Luteolin extracted from *Fridericia platyphylla* were investigated in tumor cell lines representing six different cancer tissues. Luteolin exhibited antiproliferative activity for all of the examined tumor cells, wherein U-251 glioblastoma cells were found to be the most sensitive to Luteolin [98]. They further found that Luteolin effectively inhibited the migration and tumorigenesis of U-251 cells and induced apoptosis by depolarizing the mitochondrial membrane, phosphorylating ERK proteins, cleaving PARP and Caspase-9, and promoting DNA damage by a H2A histone family member X (H2AX) phosphorylation [98].

The molecular effects of Luteolin were also investigated in relation to the epidermal growth factor (EGF)-induced proliferation of glioblastoma cells and the ability of Luteolin to induce apoptosis in these cells, as EGFR was found to be overexpressed in glioblastomas [99,100]. In their study, Anson et al., 2018 showed that Luteolin significantly decreased the proliferation of glioblastoma cells in vitro in the presence or absence of EGF, wherein Luteolin caused a decrement in the expression levels of phosphorylated Akt, mTOR, p70S6K, and MAPK in the presence of EGF. This demonstrated that Luteolin had an inhibitory effect on the downstream molecules that EGFR activates [99]. Additionally, Luteolin was found to induce cleavages of Caspase and PARP, and apart from its ability to induce apoptosis, Luteolin was identified to promote cell cycle arrest [99]. Similarly, Powe et al., 2022 showed that Luteolin combined with Erlotinib, an EGFR inhibitor, decreased proliferation and induced apoptosis in glioblastoma cell lines overexpressing EGFR or glioma cells expressing truncated EGFR. Mechanistically, the combination of Luteolin with Erlotinib was found to reduce the phosphorylation of downstream signaling molecules of EGFR, including Akt, NF kappa B, and STAT3 [100]. Furthermore, it was suggested that the effectiveness of Erlotinib might be enhanced by combining it with Luteolin, which can hold a new therapeutic strategy for glioblastoma [100].

The effect of Luteolin on the invasion and metastasis of glioblastoma cells was also investigated. It was found that Luteolin was able to inhibit the migration of U-87 MG and T98G glioblastoma cells via intervening with the PI3K/AKT activation, downregulating the expression of Cdc42. The inhibition of migration of glioblastoma cells after Luteolin treatment was found to be related to the increment in the degradation of Cdc42 protein by activating the proteasome degradation pathway [101]. Similarly, in a more recent study, Wang et al., 2017 showed that Luteolin inhibits the migration of U251MG and U87MG human glioblastoma cell lines by downregulating MMP-2 and MMP-9 and upregulating the tissue inhibitor of metalloproteinase (TIMP)-1 and TIMP-2. Luteolin was also found to inhibit phenotype related to EMT [102]. Moreover, they established that the phosphorylated insulin-like growth factor-1 receptor (p-IGF-1R)/PI3K/AKT/mTOR signaling pathway was the target of Luteolin for reducing the migration of glioblastoma cells [102].

The combination of the therapeutic effect of Luteolin with another plant-derived compound Silinib, a flavonoid from the milk thistle, on the glioblastoma cells was also examined [103,104]. Chakrabarti and Ray, 2016 showed that the combination of Luteolin and Silinib demonstrated a synergistic inhibitory effect on the human glioblastoma T98 G and U87MG cancer cell lines through different mechanisms [104]. The combination inhibited glioblastoma cells’ growth by significantly inducing apoptosis and inhibiting invasion and migration. Additionally, the combination of Luteolin and Silinib resulted in the inhibition of rapamycin-induced autophagy, which is a survival mechanism for cancer cells, by suppressing the protein kinase alpha (PKCα), an autophagy inducer, and inducing apoptosis through downregulating iNOS, apoptosis inhibitor. It also significantly increased the expression of tumor suppressor miR-7-1-3p in glioblastoma cells [104]. Furthermore, in another study by Chakrabarti and Ray, 2015, the antitumor effects of the combination of Luteolin and Silinib were demonstrated in different human glioblastoma cells, LN18 and SNB19, and in glioblastoma stem cells; the researchers found that the combination resulted in the inhibition of cell proliferation and migration and, thus, induction of apoptosis in LN18 and SNB19 cells and glioblastoma stem cells [103]. The apoptosis induction by the combination therapy was related to the inhibition of PKCα, X-linked inhibitor of apoptosis (XIAP), and iNOS. Notably, the inhibition of the cell viability via a combination of Luteolin and Silinib was found to be more effective than the conventional chemotherapy drugs, temozolomide and bischloroethylnitrosourea [103].

### 4.6. Luteolin and Liver Cancer

Luteolin was discovered to also exert anticancer properties in liver cancer by regulating different signaling pathways. In the study by Ding et al., 2014, Luteolin was demonstrated to inhibit the proliferation of SMMC-7721 and BEL-7402 liver cancer cells in a time- and dose-dependent manner, and it caused cell cycle arrest at the G1/S stage, reducing the mitochondrial membrane potential [105]. Luteolin was found to promote a higher rate of apoptosis, and the typical apoptotic morphological changes were observed in the investigated liver cancer cells. Moreover, the Luteolin application resulted in the upregulation of Bax and Caspase-3 and the downregulation of the Bcl-2 protein levels in SMMC-7721 and BEL-7402 liver cancer cells [105]. Additionally, in one of the earlier studies, Luteolin was found to induce significant cell death in HepG2 hepatocarcinoma cells and strongly reduced tumor volume in a xenograft tumor model [106]. These effects were found to be associated with Luteolin’s activation of AMP-activated protein kinase (AMPK). Luteolin was found to have a strong inhibitory effect on the NF-κB, and it was revealed that AMPK activity was essential to the inhibition of cancer cell growth, possibly through modulation of the activity of NF-κB. Moreover, Luteolin was shown to cause the release of ROS, and this intracellular ROS was suggested to mediate AMPK-NF-κB signaling in HepG2 hepatocarcinoma cells [106]. In one of the recent studies, Luteolin was shown to inhibit the Akt/osteopontin (OPN) pathway, which resulted in the promotion of caspase-dependent apoptosis in SK-Hep-2 human hepatocellular carcinoma cells [107]. OPN is a secreted glycophosphoprotein that has been identified as overexpressed in several cancer types and is involved in tumor growth, survival, and angiogenesis [108]. OPN was correlated with the inhibition of apoptosis and induction of cell proliferation in cancer cells [109]. However, the effect of Luteolin on the OPN was first revealed in a study by Im et al., 2018 [107]. Furthermore, in the research by Niu et al., 2015, the group examined the antitumor effect of Luteolin in H22 hepatoma tissue by focusing on its possible impact on angiogenesis. They revealed that Luteolin inhibited tumor angiogenesis and cell proliferation in vivo by downregulating expressions of the lymphocyte function-associated antigen 3 (LFA-3), proliferating cell nuclear antigen (PCNA), and upregulating intracellular adhesion molecule 1 (ICAM-1) expression in tumor tissue [110].

Luteolin was also identified to exhibit a synergistic effect with the chemotherapeutic agents in the hepatocellular carcinoma cells, suggesting the potential role of Luteolin in sensitization to chemotherapy. In the study of Xu et al., 2016 Luteolin was found to synergize the anticancer effects of 5-fluorouracil in the HepG2 and Bel7402 hepatocellular carcinoma cells, as cell viability and apoptosis analyses demonstrated that synergistic inhibition of growth of hepatocellular carcinoma cells was closely associated with apoptosis [111]. Furthermore, the synergistic effect of the Luteolin and the 5-fluorouracil combination was also found to be related to the increased Bax/Bcl-2 ratios and p53 levels and inducement in the cleavage of PARP [111]. Luteolin was also shown to exhibit a synergistic effect with TRAIL in liver cancer cells. Nazim and Park, 2019 demonstrated that Luteolin and TRAIL exhibited a synergistic impact in the TRAIL-resistant Huh7 human liver cancer cells. They also showed the induction of autophagic flux in the cells, and when an inhibitor attenuated the autophagic fluctuation, a significant decrease in the DR5 expression was observed [112]. Treatment of cells with the genetically modified autophagy-related 5 siRNA resulted in the abolishment of the Luteolin-mediated sensitizing effect of TRAIL. Moreover, when cells were pretreated with an inhibitor of JNK, the upregulation of DR5 expression that Luteolin induces was found to be decreased; thus, it was suggested that JNK activation induced the DR5 expression. Overall, their study concluded that Luteolin significantly increased the TRAIL-initiated apoptosis, and this effect was likely regulated by autophagy and JNK-mediated DR5 expression [112]. Cancer-targeting gene-viro therapy (CTGVT), which uses oncolytic viral vectors that encode anticancer genes, has been shown to have potent anticancer activities [113]. In a study by Wang et al., 2021, the researchers used oncolytic vaccinia virus (VV) that encoded the IL-24 gene (VV-IL-24) as the model for CTVGT in the treatment of liver cancer and investigated the potential synergistic effect of Luteolin to the treatment [114]. The study revealed that the combination of VV-IL-24 and Luteolin caused a stronger decrement in the viability and induction of apoptosis of liver cancer cells compared to Luteolin or VV-IL-24 alone. Additionally, in the MHCC97-H xenograft liver cancer model, combination treatment resulted in significant tumor reduction compared to a single treatment, exhibiting the synergistic mechanism employed by VV-IL-24 and Luteolin to treat liver cancer [114].

The effects of Luteolin in the regulation of autophagy and the possible role of autophagy in Luteolin-induced apoptosis were also investigated in several studies. In the study by Cao et al., 2017, it was found that Luteolin reduced the viability of SMMC-7721 hepatocellular carcinoma cells that express wild-type p53 and caused significant cell cycle arrest at G0/G1 phase, resulting in the dramatic apoptosis induction, as proved by the increased Caspase-8 and decreased Bcl-2 expressions [115]. When the effect of Luteolin on autophagy was investigated, it was revealed that Luteolin caused an increase in the intracellular autophagosome number, induced conversion of microtubule-associated protein 1 light chain 3 (LC3B-I) to LC3B-II, and promoted expression of Beclin-1 in SMMC-7721 cells. Additionally, treatment of Luteolin and the autophagy inhibitor resulted in the decrement of the Luteolin-induced apoptosis, suggesting that Luteolin induced apoptosis in SMMC-7721 liver cancer cells via autophagy [115]. In contrast, in the study by Lee and Kwon, 2019, Luteolin was demonstrated to induce autophagy only in the p53-null Hep3B hepatocellular carcinoma cells and discovered that Luteolin-induced autophagy inhibited the reduction in cell numbers when co-incubated with the autophagy inhibitor, meaning that autophagy induced by Luteolin in Hep3B cells resulted in the enhancement of cell viability instead of cytotoxic effects [116]. The conflicting results with the study of Cao et al., 2017 [115] were suggested to be related to the concentration of Luteolin, which was ten times higher than that which Lee and Kwon, 2019, used in their study; thus, it was propounded that the concentration is a critical determinant in the decision of whether Luteolin-induced autophagy promotes or induces apoptosis [116]. Moreover, the study by Lee and Kwon, 2019, also revealed other significant findings, such as that Luteolin was able to induce apoptosis and inhibit cell proliferation in only p53-null Hep3B cells, not in the p53-wild type HepG2 hepatocellular carcinoma cells. They also found that Luteolin caused oxidative stress and ER stress in the Hep3B cells, suggesting that Luteolin-induced ER stress might show anticancer effects in a p53-independent manner [116].

### 4.7. Luteolin and Breast Cancer

Most of the research on the anticancer properties of Luteolin has been conducted in breast cancer. The most ascertained mechanisms by which Luteolin exerts its anti-breast cancer properties were the modulation of apoptosis and angiogenesis through different signaling pathways, and Luteolin was shown to be a promising therapeutic agent for breast cancer [117]. In the study by Sui et al., 2016, Luteolin was shown to inhibit cancer cell proliferation and cause suppression in the expressions of p-STAT3, p-EGFR, p-Akt, and p-Erk1/2 in the MCF-7 breast cancer cells that EGF induces [118]. Their study demonstrated that Luteolin could suppress the EGF-induced activities of EGFR signaling in human breast cancer cell lines and suggested that STAT3, MAPK/ERK1/2, and PI3K/Akt signaling pathways are the main pathways through which Luteolin exhibits its effects on EGFR signaling [118]. Additionally, Luteolin was found to significantly inhibit the proliferation, block the development of the cell cycle, and induce apoptosis in insulin growth factor-1 (IGF-1) stimulated MCF-7 breast cancer cells [119]. Additionally, it caused a dramatic reduction in the IGF-1-dependent IGF-1R phosphorylation and phosphorylated-Akt levels without interfering with the Erk1/2 phosphorylation [119]. Further studies revealed that estrogen receptor alpha (ERα) was directly involved in the IGF-1-induced inhibitory effects of Luteolin on the cell growth that significantly reduced ERα expression, suggesting that Luteolin exhibited its inhibitory effects by inhibiting IGF-1 mediated PI3K/Akt pathway, which is dependent on the ERα expression [119]. In a recent report, Luteolin was found to induce apoptotic and necrotic cell death in MDA-MB-231 cells, and this effect was unimpaired by HIF-1 activation [120]. Luteolin was found to stimulate autophagy in these cells, but this did not contribute to the cytotoxic effects; instead, it was shown to play a protective response. Additionally, Luteolin demonstrated an induction in the decrease in HIF-1 transcriptional activity accompanied by a decrement in the stemness and invasion protein markers and the migratory capacity of breast cancer cells. As a result, the study highlighted the possible use of Luteolin in hypoxic tumors as a successful therapeutic agent [120].

The role of Luteolin in the apoptosis regulation of breast cancer cells was also mechanistically investigated. In earlier studies by Lee et al., 2012, Luteolin was demonstrated to inhibit the growth of MDA-MB-231 ER-negative breast cancer cells, which was associated with cell cycle arrest at the G2/M and S stages and induction of apoptosis [121]. Furthermore, the group revealed that the cell cycle arrest and apoptosis induction by Luteolin was related to Luteolin’s ability to decrease Akt, polo-like kinase 1 (PLK1), cyclin B1, cyclin A, CDC2, cyclin-dependent kinase 2 (CDK2) and Bcl-xL and increase p21 and Bax expressions, which are partly explained by Luteolin’s inhibitory effect on the EGFR pathway [121]. In MCF-7 breast cancer cells, Luteolin has been identified to cause cell cycle arrest at the sub-G1 and G1 phases, and it induced the expressions of death receptors such as DR5 and activated caspase signaling cascades, where it was identified to promote caspase-8/-9/-3 activities and cleavage of PARP [122]. The activation of Caspase-8 and Caspase-9 induced Caspase-3 activity was observed in the extrinsic and intrinsic pathways of apoptosis. Moreover, Luteolin was found to cause a loss of mitochondrial membrane potential, induce the release of cytochrome c, and increase Bax expression via inhibition of Bcl-2 expression. Thus, it was suggested that Luteolin induced cell cycle arrest and apoptosis by activating extrinsic and intrinsic pathways in MCF-7 breast cancer cells [122]. In a recent study by Huang et al., 2019, Luteolin was identified to suppress proliferation and cause cell cycle arrest in the S phase by downregulating cyclin D1 and Survivin expressions in MDA-MB-231 breast cancer cells in a dose-dependent manner [123]. Luteolin was found to increase the ratio of Bax/Bcl-2 and Caspase-3 levels in breast cancer cells, thereby inducing apoptosis. The study also found that Luteolin dose-dependently inhibited the telomerase levels and caused the phosphorylation of NF-κB and the target gene of NF-κB, c-Myc to suppress the human telomerase reverse transcriptase (hTERT) expression that is known to encode telomerase’s catalytic subunit [123]. Thus, it was said that in MDA-MB-231 breast cancer cells, the growth inhibition by Luteolin might be related to its ability to target hTERT [123]. Moreover, in recent research, Wu et al., 2020, showed that Luteolin caused inhibition of proliferation and induced apoptosis of ER-positive cells that are resistant to the chemotherapy agent, Tamoxifen [124]. Additionally, Luteolin was shown to induce cell cycle arrest in the G2/M phase and reduce the mitochondrial membrane potential of these cells. As Luteolin reduced the levels of activated PI3K/Akt/mTOR signaling cascade, the combination of Luteolin and PI3K, Akt, or mTOR inhibitors resulted in the synergistic increase in apoptosis in Tamoxifen-resistant ER-positive breast cancer cells [124]. Mechanistically, Luteolin was found to suppress the expressions of K-Ras, H-Ras, and N-Ras, which are the activators of PI3K, and this suppression was found to be related to the induction of expression of mixed-lineage leukemia 3 (MLL3), a tumor suppressor, by Luteolin. Further, MML3 was identified to increase the monomethylation level of Histone 3 Lysine 4 on the Ras genes’ enhancer and promoter regions, resulting in the repression of the Ras family expressions [124].

Moreover, the role of Luteolin in the metastasis and angiogenesis of breast cancer cells was comprehensively researched. In earlier studies, Luteolin caused the suppression of proliferation and significantly reduced the migration rate of MCF-7 cells by decreasing the expression levels of astrocyte-elevated gene-1 (AEG-1) and MMP-2 levels [125]. In the MCF-7 cell line, Luteolin 8-C-β-fucopyranoside, the C-glycoside of Luteolin without O-glycoside, was also reported to suppress the invasion of breast cancer cells by ERK1/activator protein-1 (AP-1) and ERK/NF-κB pathways [126]. Cook et al., 2015, demonstrated that Luteolin treatment resulted in a reduction in the viability of breast cancer cells, progestin-dependent VEGF secretion from breast cancer cells, and tumor growth in the human breast cancer xenograft model dependent on medroxyprogesterone acetate, synthetic progestin [127]. Furthermore, Luteolin caused a decrement in the xenograft tumor VEGF expression and blood-vessel density and inhibited the medroxyprogesterone acetate-induced acquisition of stem cell-like properties by breast cancer cells. Thus, the group showed that Luteolin was a potent chemotherapeutic that effectively suppressed the growth of progestin-dependent human xenograft tumors, inhibited angiogenesis, and caused a restriction in the conversion of breast cancer cells into stem cell-like cells [127].

In another study, Luteolin was identified to inhibit the migration and invasion of highly metastatic triple-negative breast cancer cell lines. It caused reversion in the EMT that was determined via alteration in the morphological characteristics, downregulation in the mesenchymal markers and EMT-related transcription factors, and upregulation in the epithelial markers [128]. In the in vivo metastasis experiment, Luteolin was found to cause significant inhibition in breast cancer lung metastasis and a decrease in the mesenchymal marker vimentin and transcription repressor Slug in the primary tumor [128]. Moreover, Luteolin was found to result in suppression of β-catenin expression in vitro and in vivo, where overexpression of β-catenin caused inhibition of the anti-invasive and antimetastatic effects of Luteolin in the breast cancer cells, suggesting that Luteolin suppressed metastasis of breast cancer via reversion of EMT that might be regulated by the β-catenin downregulation [128]. In a recent study, Cao et al., 2020 demonstrated that treatment of Luteolin caused significant inhibition of yes-associated protein (YAP)/transcriptional co-activator with PDZ-binding motif (TAZ) activity by promoting its degradation in the triple-negative breast cancer cells [129]. Additionally, Luteolin resulted in the decrement of mesenchymal and increment of epithelial markers in triple-negative breast cancer and TAZ-induced mesenchymal cells. Coherently treating Luteolin caused cell migration inhibition in triple-negative cancer cells [129]. Additionally, Luteolin was reported to inhibit the proliferation and metastasis of androgen receptor-positive triple-negative breast cancer cells by epigenetically downregulating the expression of MMP9, causing a decrement in the levels of Akt/mTOR-inducing H3K27ac and H3K56ac [130].

Furthermore, the effect of Luteolin on miRNAs in breast cancer was also researched. In their study, Sun et al., 2015, demonstrated that Luteolin could cause significant inhibition in cell survival, cell cycle, and tube formation in breast cancer cells and could also inhibit the expression of proteins and mRNAs regulated by Notch signaling pathways and associated miRNAs [131]. In particular, Luteolin caused enhancement in the miR-181a, miR-139-5p, miR-224, and miR-246 expression levels and decrement in the miR-155 level in the breast cancer cell lines (MDA-MB-231 and MCF-7 cells). When MDA-MB-231 cells were treated with the Notch-1 siRNA and miRNA mimics, miRNA levels were changed, Notch signaling-related proteins were reduced, and tumor survival, invasion, and angiogenesis were decreased, suggesting that Luteolin inhibited Notch signaling by regulating miRNAs [131]. In another study, Luteolin was identified to cause an increment in the expression of miR-203 in breast cancer cells, and it was further proved that the inhibition of breast cancer cell growth and the EMT progress in breast cancer by Luteolin was achieved through the miR-203 regulation [132]. Further mechanistic studies revealed that inhibition of Ras/Raf/MEK/ERK signaling via miR-203 by Luteolin was associated with its anti-breast cancer properties [132].

The potential synergistic effect of Luteolin with other plant-derived or chemotherapeutic agents in breast cancer cells has also been well documented. Shih et al., 2010, examined the anticancer effects of the combination of Luteolin and Quercetin, a flavonoid, on the nicotine-induced MDA-MB-231 cells, as breast cancer risk is highly associated with active and passive smoking; the breast cancer cells were induced with nicotine compound [133]. Their study found that the combination of Luteolin and Quercetin dramatically reduced the proliferation of MDA-MB-231 cells via downregulation of the α9- nicotinic acetylcholine receptor expression. Furthermore, the combination of these two flavonoids resulted in the inhibition of colony formation in nicotine-induced MDA-MB-231 cells, and the number of colonies formed was significantly reduced in α9- nicotinic acetylcholine receptor knockdown cells after the combination therapy, suggesting that anti-transforming activities induced by Luteolin or Quercetin were not restricted to specific inhibition of the α9- nicotinic acetylcholine [133]. Similarly, Yang et al., 2022, aimed to demonstrate the combined effects of the polyphenolic plant-derived compounds present in the sugarcane: Luteolin, Apigenin, Tricin, Quercetin, and p-Coumaric Acid in MCF-7 breast cancer cells [134]. The combination of Luteolin with p-Coumaric Acid demonstrated the best synergistic effect in inhibiting MCF-7 cells’ growth at all tested concentrations. In contrast, the combination of Luteolin and Apigenin at the half-maximal effective concentration (EC50) showed an antagonistic effect. However, the combination index value was calculated to decrease as the inhibition rate increased, which exhibited a synergistic effect at an inhibition rate higher than 70%. Moreover, Luteolin and Quercetin combination demonstrated an antagonistic effect at an inhibition rate higher than 50% [134]. Therefore, using Luteolin in combination with other plant-based chemicals could be a potent chemotherapeutic strategy for breast cancer treatment.

Luteolin also exhibited a synergistic effect with different chemotherapy drugs, resulting in the chemosensitizing impact in the breast cancer cells towards the chemotherapeutics being used. For instance, in earlier studies, Sato et al., 2015, demonstrated that Luteolin was able to decrease the cytotoxicity induced in the MCF-7 cells by Doxorubicin and even caused the attenuation of the Doxorubicin-induced cytotoxicity in the presence of ER antagonist and the ER-negative MDA-MB-453 human breast cancer cells. Additionally, Luteolin decreased the ROS generation induced by Doxorubicin in MCF-7 cells [135]. Similarly, in a recent study by Wu et al., 2021, Luteolin was shown to enhance the antitumor efficacy of Doxorubicin by reducing the viability, colony formation, and invasion of breast cancer cell lines, 4T1 and MDA-MB-231 [136]. Furthermore, Luteolin was also found to enhance the anticancer activity of the chemotherapeutic agent, Lapatinib, in human breast cancer cells [137]. As demonstrated by Zhang et al., 2017, the application of Luteolin and Lapatinib in combination resulted in a synergistic effect in relation to the inhibition of cell viability and apoptosis induction in BT474 cells [137]. The combination of these drugs caused the inhibition of ERBB1 and ERBB2 mRNA and protein levels. The combination also reduced the phosphorylation of Akt and ERK1/2 in BT474 human breast cancer cells, suggesting that a combination of Luteolin and Lapatinib synergistically inhibited the growth of breast cancer cells, possibly by inducing apoptosis via the deactivation of Akt and ERK signaling cascades [137].

The synergistic effect of Luteolin with Celecoxib was also demonstrated in different studies [138,139]. In a study by Jeon and Suh, 2013, the researchers showed that a combination of Luteolin and the chemotherapy agent Celecoxib caused a significant decrease in the viability of MCF-7 and MDA-MB-231 breast cancer cells compared to the application of either drug alone [138]. Additionally, the combination treatment increased breast cancer cell apoptosis, and this increment was revealed to be greater than the additive increase; combination treatment resulted in a decrement of phosphorylated Akt levels [138]. Similarly, in further studies, the synergistic effect of Luteolin and Celecoxib was shown in the inhibition of cell growth in four different breast cancer cells [139]. Mainly, combination treatment caused significant tumor growth reduction in vivo in the xenograft mouse model of MDA-MB-231 cells. The molecular mechanism of this synergistic effect of Luteolin and Celecoxib was suggested to be associated with the Akt inactivation and inhibition of ERK signaling MCF-7 and MCF7/HER18 cell lines and with the inactivation of Akt and activation of ERK signaling cascade in MDA-MB-231 and SKBR3 cells [139]. These results suggested that the synergistic effect of the combination of Celecoxib and Luteolin was possibly dependent on the ER in human breast cancer cells [139]. Another chemotherapeutic drug in which Luteolin demonstrated a synergistic effect was Paclitaxel; the combination of these drugs resulted in an increase in apoptosis compared to Paclitaxel alone in MDA-MB-231 cells, and the combination caused activation of Caspase-8 and Caspase-3 and increased Fas expression. The increment of Fas expression was considered to be related to the inhibition of STAT3 by combination therapy [140]. Similarly, in a recent study, Luteolin was further shown to enhance the cytotoxicity of Paclitaxel in MDA-MB-231 breast cancer cells [141]. Overall, it can be said that using Luteolin in combination with known and in-use chemotherapy drugs can improve the therapeutic outcome, and there is a need to test these combinations further in clinical settings.

**Table 1:** Summary of key findings regarding the anticancer properties of Luteolin in different cancer types. Arrows represent (↑) upregulation and (↓) downregulation of molecules.

Cancer Type	Model System (Cell Lines or Animal Models)	Key Molecular Target(s) or Signaling Pathway(s)	Effects	Reference
Colon	Azoxymethane-induced mice	↓ MMP-2/-9	Inhibition of metastasis	[43]
	HCT-15 cells	↓ Wnt/β-Catenin/GSK-3β signaling cyclin D1 and Bcl-2 levels, ↑ Bax and Caspase-3 levels	Inhibition of proliferation, induction of apoptosis, and G2/M cell cycle arrest	[45]
	HT-29 and SNU-407 cells	↑ Nrf2 levels and interaction between Nrf2 and p53	Induction of apoptosis	[48]
	LoVo cells	↓ CDC2 and Cyclin B levels ↑cytochrome c- and dATP-mediated activation of APAF-1	G2/M cell cycle arrest Induction of apoptosis	[50]
	HT-29 cells	↑ miR-384 levels	Inhibition of migration and invasion	[55]
Lung	A549 cells	↑ MEK/ERK signaling pathway	Inhibition of migration and induction of apoptosis	[57]
	NCI-H460 cells	↑ peIF2-α and CHOP levels Accumulation of LC3 II protein and ↑ LC3 puncta levels	Induction of ER stress-mediated apoptosis Induction of autophagy	[57]
	NCI-H1975 and NCI-H1650 cells Patient-derived xenograft mouse model	↓ LIMK1 signaling pathway	Inhibition of proliferation and anchorage-independent cell growth Inhibition of tumor growth	[59]
	A549 cells	↓ Src/FAK and its downstream Rac1, Cdc42, and RhoA pathways	Inhibition of invasion and metastasis	[71]
	Vascular endothelial cells of NSCLC	↑ miR-133a-3p/PURB- mediated MAPK and PI3K/Akt pathways	Inhibition of migration and invasion	[74]
	H460 and A549 cells Mice xenograft models of lung cancer	↓ circ_0000190 levels and ↑ miR-130a-3p (target of circ_0000190)	Inhibition of cell viability, migration, invasion, and colony formation and induction of apoptosis Inhibition of tumor growth	[75]
Prostate	PC-3 cells Xenograft prostate tumor model	↓ VEGFR-2-regulated AKT/ERK/mTOR/P70S6K/MMPs pathway	Inhibition of cell viability, migration, and invasion Inhibition of tumor growth and angiogenesis	[78]
	PC-3 cells	Inhibition of Wnt signaling by ↑ FZD6 levels	Inhibition of prostate cancer stemness	[79]
	LNCaP cells	↑ Prostate-derived Ets factor (PDEF) levels	Inhibition of proliferation and invasion	[80]
	PC3 and LNCaP cells	↓ miR-301 levels	Inhibition of proliferation and induction of apoptosis	[85]
Gastric	BGC-823 gastric carcinoma xenografts	↓ VEGF-A and MMP-9 expressions	Inhibition of tumor growth	[87]
	MKN45 and SGC7901 cells cMet-overexpressing Patient-derived human tumor xenograft models	↓ cMet/Akt/ERK signaling	Inhibition of invasiveness and induction of apoptosis	[88]
	Hs-746T and MKN28 cells Mice xenograft model of gastric cancer	↓ Notch1 signaling	Inhibition of proliferation and migration, and induction of apoptosis Inhibition of tumor growth and induction of apoptosis	[89]
	Hs-746T cells	↓ Notch1-VEGF signaling	Inhibition of angiogenesis and vasculogenic mimicry formation	[90]
	BGC-823 and SGC-7901 cells	↑ miR-34a levels	Induction of apoptosis	[92]
Glioblastoma	U87MG and T98G cells	↓ Cdc42 expression and PI3K/Akt activity	Inhibition of migration of glioblastoma cells	[101]
	U251MG and U87MG cells	↓ p-IGF-1R/PI3K/AKT/mTOR signaling pathway	Inhibition of migration of glioblastoma cells and reduction of the EMT process	[102]
Liver	HepG2 cells	↑ AMPK signaling pathway and ROS release	Induction of cell death	[106]
	SK-Hep-1 cells	↓ Akt/ osteopontin pathway	Induction of caspase-dependent apoptosis	[107]
	SMMC-7721 cells	Induction of LC3B-I conversion to LC3B-II, and ↑ Beclin 1 expression	Induction of apoptosis, partially via autophagy	[115]
Breast	MCF-7 cells	↓ EGFR signaling via mediation of PI3K/Akt, MAPK/Erk1/2 and STAT3 signaling pathways	Inhibition of cell proliferation induced by EGF	[118]
	MDA-MB-231 cells	↓ EGFR signaling	Induction of cell cycle arrest at the G2/M and S stages and apoptosis	[121]
	MCF-7 cells	Activation of Caspase-8 and Caspase-9 induced caspase-3 activity, ↑ Bax expression by ↓ Bcl-2 expression	Induction of apoptosis by activating the extrinsic and intrinsic pathways	[122]
	MDA-MB-231 cells	↓ Human telomerase reverse transcriptase (hTERT) expression	Induction of cell cycle arrest at the S phase and apoptosis	[123]
	Tamoxifen-resistant MCF-7 cells	↑ MLL3 expression	Induction of apoptosis through H3K4 monomethylation and suppression of the PI3K/AKT/mTOR pathway	[124]
	MDA-MB-231 cells	↓ β-catenin expression	Inhibition of metastasis by reversing EMT	[128]
	MDA-MB-231 and 4T1 cells Mice xenograft model of breast cancer	↓ YAP/TAZ activity	Inhibition of EMT and migration Inhibition of tumor growth	[129]
	BT-20 and MDA-MB-231 cells	↓ MMP-9 expression through ↓AKT/mTOR-inducing H3K27Ac and H3K56Ac	Inhibition of proliferation and migration	[131]
	MCF-7 and MDA-MB-453 cells	↑ miR-203 levels and ↓ Ras/Raf/MEK/ERK signaling pathway	Inhibition of breast cancer cell growth and EMT progress	[132]

## 5. Nanodelivery Systems for Luteolin in Cancer Treatment

As stated earlier, the poor solubility of Luteolin in water causes poor bioavailability and constitutes a considerable obstacle to its therapeutic efficacy in cancer treatment [142]. To solve issues related to the poor water solubility of Luteolin, scientists have begun to formulate, design, and test the nanodelivery systems for the encapsulation of Luteolin as nanoformulations, which offer to improve the solubility of drugs, as well as to enhance their pharmacokinetic and pharmacodynamic activities; they can also help to target the therapeutic agent to cancerous tissues [143,144]. Regarding the problems mentioned earlier, Zheng et al., 2017, formulated and designed monomethyl poly(ethylene glycol)-poly(ε-caprolactone) (MPEG-PCL) micelles and loaded Luteolin into these micelles to improve the solubility of Luteolin, as well as investigating the in vivo and in vitro anticancer effect of Luteolin-loaded MPEG-PCL micelles on glioblastoma [145]. They found that spherical Luteolin/MPEG-PCL micelles were dispersible in normal saline and resulted in sustained release of Luteolin in vitro. Additionally, Luteolin/MPEG-PCL micelles demonstrated significantly higher cytotoxicity and higher promotion of apoptosis in C6 and U87 glioblastoma cells compared to free Luteolin in vitro, and further analysis revealed that downregulation of pro-Caspase-9 and Bcl-2 and upregulation of cleaved Caspase-9 and Bax proposed that Luteolin induced apoptosis via the mitochondrial pathway in vitro [145]. Furthermore, in the zebrafish and mice xenograft model, Luteolin/MPEG-PCL micelles enhanced the reduction in tumor growth, demonstrating the antiglioma activity of the Luteolin/MPEG-PCL in vivo, while Luteolin/MPEG-PCL micelles induced apoptosis more in vivo compared to free Luteolin and inhibited the neovascularization in tumor tissues [145]. In a more recent study, the anticancer effects of folic acid-modified poly(ethylene glycol)-poly(e-caprolactone) (Fa-PEG-PCL) and MPEG-PCL micelles that were loaded with Luteolin were investigated in glioblastoma cells in vivo and in vitro. As a result, the study demonstrated that Lut/MPEG-PCL and Lut/Fa-PEG-PCL micelles caused significant inhibition in cell growth and induced more apoptosis of GL261 cells compared to free Luteolin in vitro and in vivo [146]. Moreover, safety assessments showed that Lut/MPEG-PCL and Lut/Fa-PEG-PCL had no noticeable side effects in mice [146].

Different formulations have been conducted to deliver Luteolin to breast cancer cells in studies related to the delivery systems of Luteolin and breast cancer [147,148,149]. For instance, in earlier studies, Sabzichi et al., 2014, designed Luteolin-loaded phytosomes to enhance the bioavailability of Luteolin and passive targeting in breast cancer cells and improve the efficacy of Doxorubicin treatment [147]. As a result, they found that when MDA-MB-231 cells were cotreated with nanophytosomes, including Luteolin and Doxorubicin, the highest percentage of cell death was observed. Additionally, Luteolin-loaded nanoparticles resulted in a significant reduction in the Nrf2 levels compared to Luteolin alone. The expressions of the downstream genes of Nrf2, Ho1, and MDR1 were also reduced, where inhibition of Nrf2 expression significantly increased the cell death of breast cancer cells [147]. In a more recent study, Kollur et al., 2021, suggested using Luteolin-fabricated zinc oxide nanoparticles (ZnONPs) to deliver Luteolin effectively [148]. They found that Luteolin-capped ZnONPs resulted in significant anticancer activity against the MCF-7 breast cancer cells compared to Luteolin and ZnO alone. When this anticancer activity was evaluated mechanistically, it was suggested that the anticancer activity of Luteolin-ZnONPs was possibly mediated by PLK1 proteins, as shown by in silico methods [148]. Furthermore, Altamimi et al., 2021, aimed to use Luteolin-loaded elastic liposomes for the transdermal delivery for the control of breast cancer; they revealed that the elastic liposome carrier increased the drug release compared to rigid liposome and free Luteolin but also demonstrated increased permeation parameters across the rat skin [149]. Additionally, Luteolin-loaded elastic liposomes significantly inhibited the growth of MCF-7 cells, and the elastic liposome system resulted in enhanced cellular internalization to achieve the increased inhibition effect compared to the free Luteolin [149]. Thus, the elastic liposomes could be a promising strategy for the transdermal delivery of Luteolin and could improve the therapeutic efficacy of Luteolin in breast cancer treatment [149].

Furthermore, Ding et al., 2020 also designed a delivery system for Luteolin to enhance its therapeutic efficacy in gastric cancer cells. In their study, they developed Her-2-poly (lactic-co-glycolic acid) (PLGA) nanoparticles (NPs) and loaded them with Luteolin to determine the targeted inhibitory effect of the system on gastric cancer cells [150]. As a result, they observed that the Luteolin-loaded Her-2-PLGA NPs caused a significant increase in the uptake of Luteolin by SGC-7901 gastric cancer cells compared to the non-targeted microspheres. Additionally, the Luteolin-loaded Her-2-PLGA NPs dramatically inhibited gastric cancer cells proliferation and migratory ability and caused an increment in the mRNA and protein levels of forehead box protein O1 (FOXO1) [150].

## 6. Conclusions

Luteolin, as a flavonoid, has potent anticancer properties in various cancer types. It regulates multiple processes, including cell proliferation, cell cycle progression, apoptosis, angiogenesis, and migration of cancer cells, by upregulating or downregulating several critical proteins involved in these diverse signaling pathways. As discussed throughout the data in the current review, the molecular signaling cascades associated with the anticancer effects of Luteolin are dependent on the types of cancer cells or tissues, on the concentrations used in different assays, and even on the form of Luteolin. Thus, comparing and classifying according to a change in one specific metabolite or the expression level of one particular protein is challenging. Therefore, systemic approaches, including metabolomics and proteomics, may help gain a global insight into the biological and physiological processes mediated by Luteolin.

Furthermore, Luteolin has been demonstrated to enhance the anticancer effects of conventional chemotherapy agents when co-administered. Additionally, when Luteolin was combined with other flavonoids, more potent anticancer effects were observed in various cancer cells, suggesting the potential therapeutic benefits of the combination of flavonoids for cancer therapy. Further studies on Luteolin need to be conducted to enhance its targeted and efficient delivery to cancer tissues using nanotechnological strategies. Moreover, the safety profile of Luteolin for humans must be investigated in detail and fully established to implement the promising anticancer properties of Luteolin in the treatment of cancer. As a natural compound, Luteolin can open up a new therapeutic methodology, considering the toxicity experienced during conventional chemotherapeutics.

## Data Availability

Not applicable.
